# Prevalence of Heterotypic Tumor/Immune Cell-In-Cell Structure In Vitro and In Vivo Leading to Formation of Aneuploidy

**DOI:** 10.1371/journal.pone.0059418

**Published:** 2013-03-28

**Authors:** Yu-hui Chen, Shan Wang, Mei-fang He, Yanyi Wang, Hua Zhao, Han-yu Zhu, Xiao-min Yu, Jian Ma, Xiao-juan Che, Ju-fang Wang, Ying Wang, Xiao-ning Wang

**Affiliations:** 1 The State Key Laboratory of Kidney, Beijing/Provincial Key Laboratory of Biotechnology, Institute of Life Sciences, Chinese PLA General Hospital and SCUT, Guangzhou, China; 2 Shanghai Institute of Immunology, Shanghai Jiaotong University School of Medicine, Shanghai, China; 3 School of Life Sciences, Fudan University, Shanghai, China; 4 The Institute of Molecular Immunology, Southern Medical University, Guangzhou, China; Duke University Medical Center, United States of America

## Abstract

Cell-in-cell structures refer to a unique phenomenon that one living cell enters into another living cell intactly, occurring between homotypic tumor cells or tumor (or other tissue cells) and immune cells (named as heterotypic cell-in-cell structure). In the present study, through a large scale of survey we observed that heterotypic cell-in-cell structure formation occurred commonly *in vitro* with host cells derived from different human carcinomas as well as xenotypic mouse tumor cell lines. Most of the lineages of human immune cells, including T, B, NK cells, monocytes as well as *in vitro* activated LAK cells, were able to invade tumor cell lines. Poorly differentiated stem cells were capable of internalizing immune cells as well. More significantly, heterotypic tumor/immune cell-in-cell structures were observed in a higher frequency in tumor-derived tissues than those in adjacent tissues. In mouse hepatitis models, heterotypic immune cell/hepatocyte cell-in-cell structures were also formed in a higher frequency than in normal controls. After *in vitro* culture, different forms of internalized immune cells in heterotypic cell-in-cell structures were observed, with one or multiple immune cells inside host cells undergoing resting, degradation or mitosis. More strikingly, some internalized immune cells penetrated directly into the nucleus of target cells. Multinuclear cells with aneuploid nucleus were formed in target tumor cells after internalizing immune cells as well as *in situ* tumor regions. Therefore, with the prevalence of heterotypic cell-in-cell structures observed, we suggest that shielding of immune cells inside tumor or inflammatory tissue cells implies the formation of aneuploidy with the increased multinucleation as well as fine-tuning of microenvironment under pathological status, which may define distinct mechanisms to influence the etiology and progress of tumors.

## Introduction

The phenomenon of cell-in-cell structure formation, in which viable cells are internalized into other cells, has been observed for nearly one hundred years when Eberth has observed lymphocytes within intestinal epithelial cells in 1864 [1]. It occurs between either homotypic cells in which one target cell is internalized into a host cell of the same cell type [2], or heterotypic cells in which one target cell is internalized into a host cell of different cell types. This unique cell biological structure has aroused great interests in that with the long-history observation of cell-in-cell structure, it is still unclear what this cellular behavior represents under physiological or pathological status [3]. Focusing on the homotypic cell-in-cell structures, Brugge and his colleagues described a non-apoptotic cell death pathway termed “entosis” [4]. Different from phagocytosis targeting dead cells or cannibalism with no selection for dead or live cells [5], entosis is an invasive process by homotypic living cells. The internalized cells are mostly enveloped by plasma membrane in which these cells remain viable or undergo mitosis for certain period before being released to the outside of the host cells. Under some circumstances, the internalized cells undergo cell death mediated through degradation via lysosomal enzymes. Recently, Krajcovic *et al*. has provided direct evidence that the formation of homotypic cell-in-cell structure (entosis) leads to the cytokinesis failure and subsequent aneuploidy, which might contribute to revealing the roles of entosis on tumorigenesis [6].

Besides aforementioned homotypic cell-in-cell structures, the formation of heterotypic cell-in-cell structure was first observed by Humble in 1956 [7]. He described the movement of lymphocytes inside malignant cells, cells under mitosis, or megakeryocytes in bone marrow, named as emperipolesis (“inside round about wandering”). From then on, heterotypic internalization of immune cells were reported in non-neoplastic or neoplastic host cells either *in vivo* or *in vitro* [8]. We have also observed heterotypic interactions three decades ago when we studied the natural killer (NK) cell biology [9]. We have demonstrated that emperipolesis of NK cells inside tumor cells was a key step to trigger NK cell-mediated tumor cell death. In some cases, the NK cells enter the target cells in an active way but disintergrated with target cells, in turn leading to an “intracellular suicide” model of NK cells against tumor cells. The studies by Wang et al. furthermore, reported the cell death pathway of internalized NK cells inside the tumor cells. They revealed that internalization process required the actin cytoskeletal regulator ezrin while the death of NK cells inside was a typical apoptotic pathway triggered by the activation of caspase-3, which was distinct from entosis [10] or cannibalism. Makoto Takeuchi *et al.* also reported that HOZOT cells, a type of cytotoxic regulatory cells, actively penetrated into target tumor cells. It is proposed that HOZOT cells within tumor cells may exert a cytotoxic effect against the target cells partially via similar caspase-3 dependent pathway [11]. These results indicate that heterotypic cell-in-cell structures exhibit distinct biological characteristics and significance compared to entosis or cannibalism. Considering the previous studies on the heterotypic cell-in-cell structure with limited cell types, it is well worth performing a more extended survey *in vitro* and *in vivo* to elucidate the biological characteristics of this phenomenon.

The interaction between tumor cells and immune cells during heterotypic cell-in-cell structure formation observed arouses new questions as what the physiological significance is for this phenomenon. It is widely accepted that tumors escape from immune surveillance through several intrinsic mechanisms, including the weak immunogenicity of tumor antigens [12], down-regulation of major histocompatibility complex (MHC) molecules on tumor cells[13]–[14], defects of antigen processing machinery [13], [15] or the release of immuno-suppressive molecules[16]–[17]. With the observation of heterotypic cell-in-cell structures in tumors[18]–[19], it is possible that lymphocytes infiltration into tumor regions facilitates the direct cell-cell contact for the formation of heterotypic cell-in-cell structures described here. The formation of heterotypic cell-in-cell structure *in vitro,* to some extend, recapitulates the cellular behaviors occurring in tumor microenvironment *in vivo*. To elucidate the properties of heterotypic cell-in-cell structures thus might provide new clues to decipher novel cellular mechanisms of tumor escaping from immune attack as well as its roles on tumor progression.

In the present study, we intend to investigate the prevalence and characteristics of heterotypic cell-in-cell structure formation through a large scale of survey by using carcinoma cell lines from diverse tissues and immune cells from several lineages. We will also explore the biological significance of heterotypic cell-in-cell structures in pathological status, speculating the important roles of this phenomenon on the progression and prognosis of diseases.

## Materials and Methods

### Cell Lines

Tumor cell lines MCF-7, SK-BR-3, MDA-MB-231, HTB-20, Bcap-37, MDA-MB-453, MDA-MB-435 S, MDA-MB-468, PLC/PRF/5, HepG-2, Huh7, U-87.

A431, 143B, K562, CCRF, MOLT-4, Raji, HL-60, U937, B16F10 and NIH/3T3 were purchased from American Type Culture Collection (Manassas, USA). HCCLM3, RD, QGY7703, HCCC9810, PC3, BxPC-3, CL1, LO2, Chang liver were kindly gifted by Prof. Ya-jun Guo (The Second Military Medical School, China). Cells were maintained routinely in DMEM complete medium with 10% fetal bovine serum (FBS) and 100 µg/mL penicillin-streptomycin (Invitrogen, USA). NK92MI cell line was gifted by Dr Hai-ming Wei (University of Science & Technology of China, China) who bought it from ATCC and maintained in MEM-alpha medium with 15% FBS (Invitrogen, USA) supplemented with 100 µg/mL penicillin-streptomycin (Invitrogen, USA). The stem cells were gifted by Cyagen Biosciences Inc (Guangzhou, China) and maintained as a subconfluent monolayer in low glucose DMEM medium (Invitrogen, USA) with 10% FBS (Hyclone, USA) and 100 µg/mL penicillin-streptomycin (Invitrogen, USA) at 37°C with 5% CO_2_.

### In vitro Cell Internalization Assays

Target tumor cell suspension was stained with 2.5 µM CellTracker Green dye (Invitrogen, USA) for 30 min at 37°C in the absence of serum and incubated for 4 hrs with equivalent number of immune cells pre-stained with 2.5 µM CellTracker Red dye (Invitrogen, USA). The mixture of target and immune cells was cytospun onto slides in a Cytocentifuge 7620 cellspin (Wescor, USA) at 500 rpm for 3 min. DNA was stained with DAPI (Sigma-Aldrich, USA) for determination of cell internalization. Coverslips were supported on slides by grease pencil markings and mounted in Vectashield media (Vector Laboratories, USA). The percentages of cell-in-cell structures were calculated by counting 400 target cells with a double-blind method. Images were taken with a Leica AF7000 fluorescence microscope (Leica, Germany) using a 63×1.3 numerical aperture PlanApo objective.

In some experiments, adherent tumor cell lines were trypsinized and seeded into 48-well plates with 30% confluence. After overnight’s culture, the target tumor cells were incubated with immune cells at a ratio of 1∶1 for the indicated times. After incubation, the cell mixture was washed with cold PBS and fixed with PBS containing 2.5% glutaraldehyde for 20 min. After stained with Wright-Giemsa staining dye (Sigma-Aldrich, USA), cells were observed and counted under the light microscope.

### Cell-in-cell Structures in Human Tumor Tissues and Murine Flunimant Hepatitis

Tumor tissue arrays were purchased from Shanghai Biochip Company (Shanghai, China). Tissue sections of murine flunimant hepatitis models [20] were gifted by Professors Zhigang Tian and Haiming Wei from University of Science and Technology of China. Hematoxylin-eosin staining was performed by using a conventional protocol. Percentage of heterotypic cell-in-cell structure was evaluated among 100 cells in one field of light microscope. The average percentage from 5 randomly selected fields was used to represent the levels of cell-in-cell structure formation. Tumor/immune cell-in-cell structures in tissue arrays were compared between carcinoma and paired adjacent specimen. Hepatitic/immune cell-in-cell structures from control and flunimant hepatitis mice were also compared. P value was calculated by using *t-test* statistic analysis.

### Time-lapse Microscopy

Time lapse microscopy was performed as described before [10]. Briefly, cells were grown as monolayer on 35 mm dishes. Human hepatoma cell line PLC/PRF/5 was labeled with CellTracker Green BODIPY (Invitrogen, USA) while effector NK cell line NK92MI was labeled with Cell-Tracker Red CMTPX (Invitrogen, USA) according to the manufacturer’s manual. Fluorescence and differential interference contrast (DIC) or phase contrast images were obtained every 5 min for the indicated time courses with a Leica AF7000 fluorescence microscope (Leica, Germany).

## Results

### Heterotypic Cell-in-cell Structure Formation Occurs Commonly between Tumor Cell Lines and Immune Cells after in vitro Co-culture

In the previous study [10], we have observed the internalization of NK cell line NK92MI into tumor cell line A431, leading to a caspase-3 dependent apoptotic cell death. To explore how frequently this phenomenon occurred between tumor cell lines and immune cells, we have performed a large scale of survey on the formation of heterotypic tumor/immune cell-in-cell structure by using panels of tumor cell lines and immune cell lines *in vitro*. As listed in Table 1, it is found that heterotypic cell-in-cell structures were formed commonly. The most widely used cell lines in the study of cell-in-cell structure, A431 or MCF-7, underwent not only certain frequency of homotypic cell-in-cell structure formation, but were also invaded by immune cells including NK cell line (NK92MI), B cell line(Raji) and T cell lines (CCRF and Molt-4) with much more efficacy. All cell lines tested from normal tissues did not undergo homotypic cell-in-cell structure formation. But some of them (CL1 and MCF-10A) still formed heterotypic cell-in-cell structures with different types of immune cells whereas some (L02 or HUVEC) did not. Interestingly, myeloid cell lines such as HL-60 or U937 were largely exclusive for the formation of heterotypic cell-in-cell structures with target tumor cells.

**Table 1:** Heterotypic cell-in-cell structures formed between immune cell lines and tumor or normal tissue-derived cell lines.

Type of target cells		Homotypic	NK92MI	CCRF	Raji	MOLT-4	HL-60	U937
ectoderm	AC	±	+	+	++	+	−	±
	U251	−	±	±	±	±	−	−
	U87	−	++	−	−	±	−	−
	A431	+	+++	+++	+++	+++	±	+
mesoderm	143B	−	+	+	±	+	-	±
	RD	−	++	±	±	+	-	-
	K562	−	+	+	+	+	+	+
endoderm	MCF-7	+	+++	+++	+++	+++	±	+
	SK-BR-3	−	++	+	+	+	+	+
	MDA-MB-231	−	+	+	+	+	−	−
	HTB-20	−	+	+	+	+	−	−
	Bcap-37	−	+	−	±	−	−	−
	MDA-MB-453	−	−	−	−	−	−	−
	MDA-MB-435S	−	±	+	±	+	-	±
	MDA-MB-468	−	−	−	−	±	±	±
	PLC/PRF/5	±	+++	+++	+++	+++	±	++
	HepG2	−	++	++	+	+	−	−
	Huh-7	±	+	+	++	++	−	±
	HCC-LM3	±	+	+	+	+	±	+
	QGY-7703	−	+	+	+	+	−	−
	HCCC-9810	−	±	++	++	++	−	−
	PC-3	−	+	+	++	++	−	−
	BxPC-3	±	+	+	++	++	±	±
Normal cell	CL1	−	±	+	+	++	−	−
	LO2	−	±	−	−	±	−	−
	MCF-10A	−	+	+	+	+	−	−
	HUVEC	−	±	−	−	±	−	−

Notes:

The symbol “−”means that the cell-in-cell index is 0%; The symbol “±”means that the cell-in-cell index is smaller than 1%; The symbol “+” meaning that the cell-in-cell index is between 1% and 5%; The symbol “++” means that the cell-in-cell index is between 5% and 10%; The symbol “+++” means that the cell-in-cell index is between 10% and 15%;

All these results are based on more than three repeated experiments.

**Figure 1:**
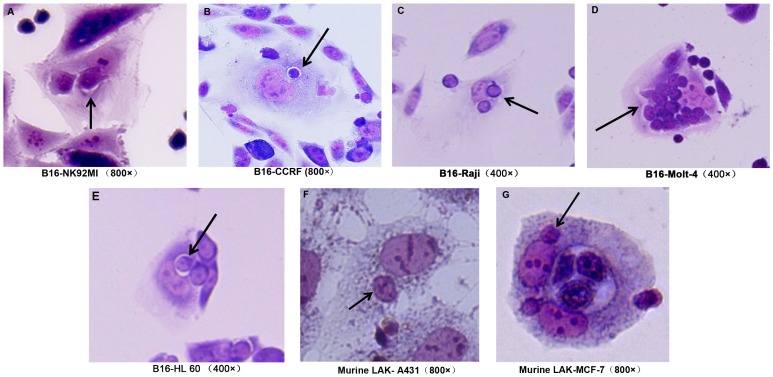
Xenotypic cell-in-cell structures between mouse tumor cells and human immune cells. Mouse skin melanoma cell line B16 cells were incubated with human immune cells, including NK, B, T and myeloid cell lines for four hours (A to E). Mouse LAK cells were incubated with human A431 and MCF cells for four hours (F to G). The cell mixture were fixed on slides by cytospin and stained with H&E dyes. (A): B16-NK92MI (B): B16-CCRF (C): B16-Raji (D): B16-MOLT-4 (E): B16-HL-60 (F): Murine LAK-A431; (G): Murine LAK-MCF-7.

When we extended our survey scope from congeneric cell-in-cell structure formation to xenogeneric one, we found that mouse tumor cell lines, such as mouse skin melanoma B16F10 (B16), was able to internalize human immune cells tested including NK cells, B cells and T cells (Table 2, Fig. 1). Mouse embryonic fibroblast NIH/3T3 cells, however, could not form heterotypic cell-in-cell structures with immune cells. It is reported that NIH/3T3 cells act as feeder cells to support the growth of primary cells with weak oncogenenicity. Considering the observation of congeneric cell-in-cell structure, the malignancy of cell lines might influence the formation of heterotypic cell-in-cell structures.

**Table 2:** Xenogeneic cell-in-cell structure formation.

	Immune Cells				
Host Cells	Homotypic	NK92MI	CCRF	Raji	MOLT-4
B16	−	+	++	++	++
NIH/3T3	−	±	−	−	−

Notes:

The symbol “–”means that the cell-in-cell index is 0%; The symbol “±”means that the cell-in-cell index is smaller than 1%; The symbol “+” meaning that the cell-in-cell index is between 1% and 5%; The symbol “++” means that the cell-in-cell index is between 5% and 10%; The symbol “+++” means that the cell-in-cell index is between 10% and 15%;

All these results are based on more than three independent experiments.

In addition, we also observed that mouse lymphokine-activated killer cells (LAK cells) penetrated human epidermal carcinoma cell line A431 and breast carcinoma cell lines MCF-7 (Fig. 1F and Fig. 1G). In the previous survey, two cell lines underwent both homotypic and heterotypic congeneic cell-in-cell structures with high frequency. They also displayed strong capacity in xenogeneic cell-in-cell structure formation, which suggested that cell-in-cell structure formation represents one of conserved biological processes during evolution.

### Poorly Differentiated Cells are Capable of Internalizing Immune Cells with Comparable Frequency to Tumor Cell Lines

**Figure 2:**
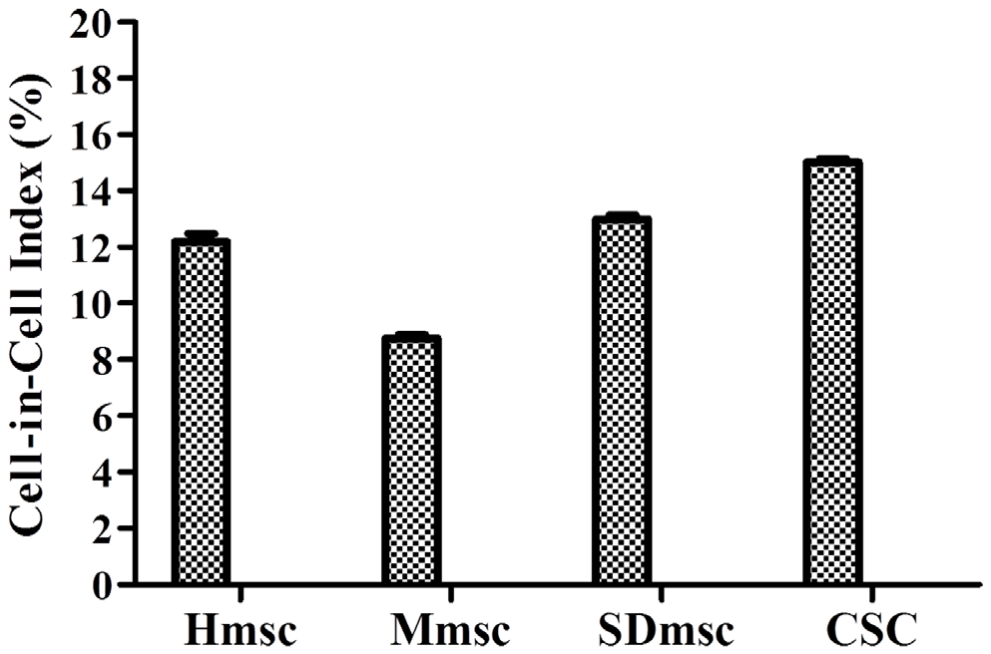
Poorly differentiated cells are capable of internalizing immune cells with similar frequency. Mesenchymal stem cells (MSCs) from human, mouse and Sprague-Dawly rat and human cancer stem cells (CSCs) were used to form heterotypic cell-in-cell structures with NK92MI for four hours. The cell-in-cell index was calculated as indicated in “methods and materials”. Three independent experiments were performed for statistic analysis.

We further used poorly differentiated stem cells to observe the formation of heterotypic cell-in-cell structures. Mesenchymal stem cells (MSCs) are multi-potent stem cells that can differentiate into a variety of cell types, including osteoblasts, chondrocytes and adipocytes *in vitro* [21]. These cells also display immune modulation that make them as one of the potential ways for the treatment of certain autoimmune diseases [22]. Considering their immune regulatory roles, we intended to determine if these cells were able to form heterotypic cell-in-cell structures when they encountered immune effector cells as well. When incubating NK92MI cells with three stem cell lines from human, mouse or Sprague-Dawley(SD) rat, we observed heterotypic cell-in-cell structure formation whereas no homotypic MCS cell-in-cell structures occurred (Fig. 2), suggesting that internalization of effector immune cells by MSCs might act as one of mechanisms to modulate immune responses, either re-educating or eliminating them afterwards. This phenomenon, therefore, might fine-tune immune microenvironment for the amelioration of pathological syndromes. Furthermore, when incubating cancer stem cells (CSCs) with lymphocytes, we found that heterotypic cell-in-cell structures were formed with slightly higher frequency.

### Hepatitis Inflammation Promotes the Formation of Heterotypic Hepatic/immune Cell-in-cell Structures in vivo

**Figure 3:**
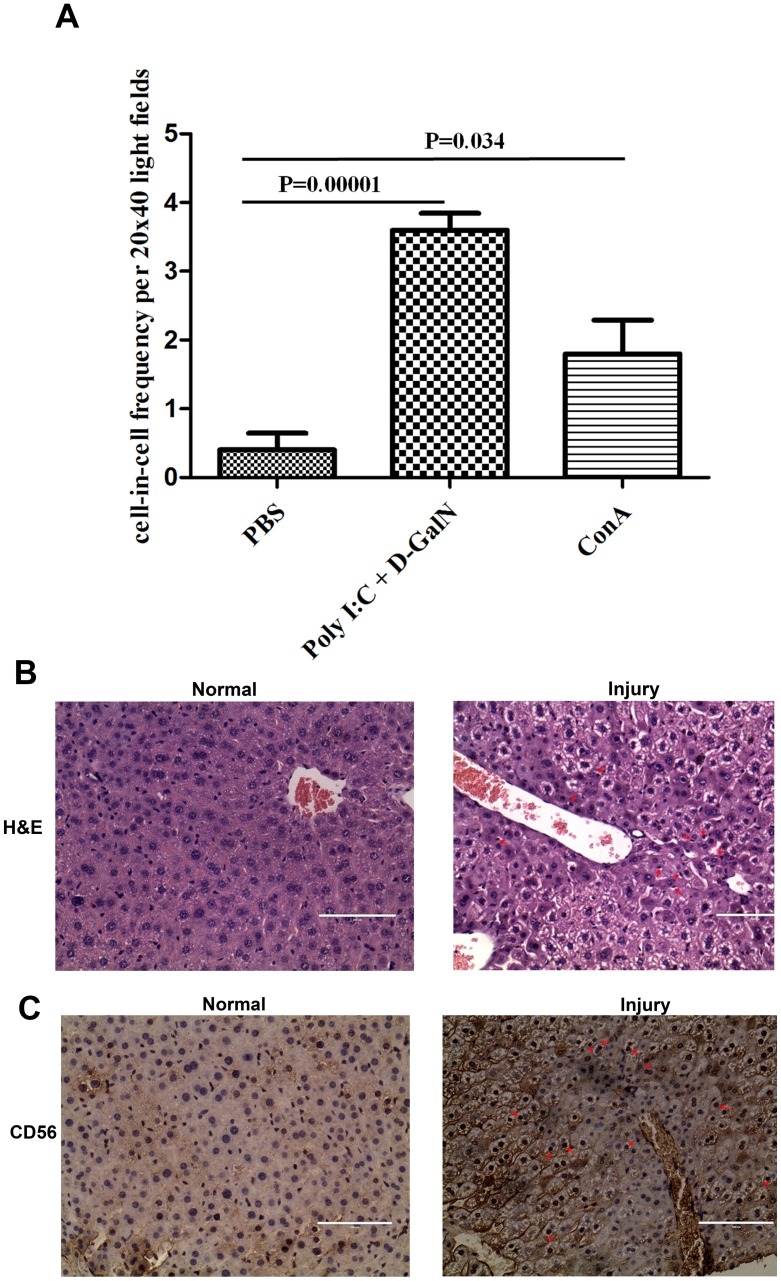
Heterotypic cell-in-cell structures in murine fluminant hepatitis specimen. (A): Statistics results of heterotypic cell-in-cell structures in murine fluminant hepatitis liver. (B): Giemsa staining of liver tissues. Left: Normal liver section; Right: injury liver section (Red arrows: heterotypic cell-in-cell structures) (C): NK cell staining of liver tissues. Left: Normal liver section; Right: injury liver section (Red arrows: NK cells in heterotypic cell-in-cell structure) Scale bar: 100 µM.

Infiltration of immune cells into tissues occurs frequently under inflammation. As the formation of heterotypic cell-in-cell structure occurs widely after *in vitro* co-culture, we intend to further investigate whether this phenomenon will be observed under physiological or pathological status *in vivo*. By using two acute mouse fluminant hepatitis models established through co-injection of Poly I:C and D-GaIN [20]or ConA *i.v*. [23], we have detected the frequency of heterotypic cell-in-cell structure formation in hepatitis inflammatory liver tissues from two models. More heterotypic cell-in-cell structures were formed in livers of two hepatic mouse models than in control mice (p = 0.00001 and p = 0.034, respectively) (Fig. 3A). With more severity of hepatitis in mouse model induced by Poly I:C and D-GaIN, the formation of heterotypic cell-in-cell structures increased dramatically than that in ConA-induced mouse model (3.6±0.6/field *vs* 1.8±1/field). Looking closely at the morphology of *in situ* heterotypic cell-in-cell structures (Fig. 3B), internalized lymphocyte cells were mostly NK cells dominant in liver (Fig. 3C) and enveloped by the membrane in the cytosol and disintergrated with the host cells (red arrow in Fig. 3B). They maintained intact nucleus morphology while host and adjacent liver cells maintained intact cell structure. Our results for the first time demonstrated the formation of heterotypic cell-in-cell structure with a higher incidence under inflammatory status than normal one.

### Heterotypic Tumor/immune Cell-in-cell Structures are Present in situ with a Higher Frequency

**Figure 4:**
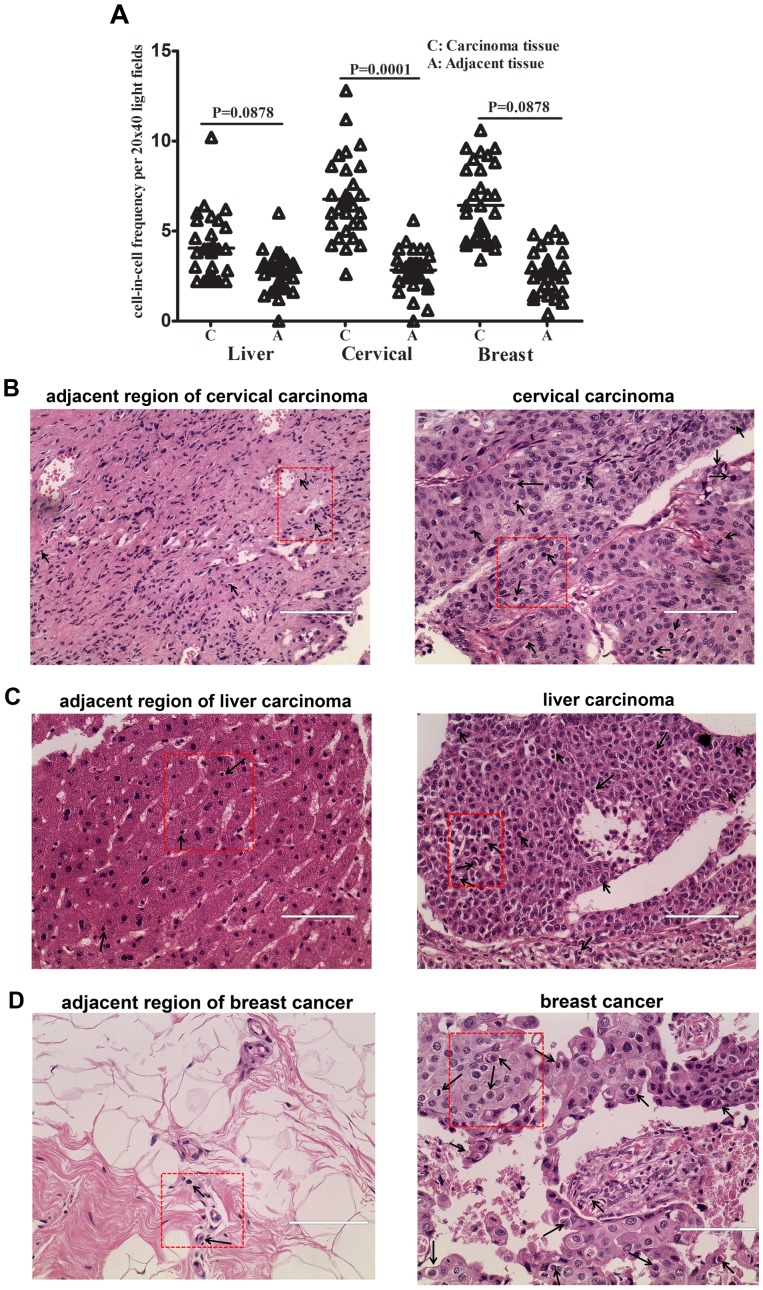
Heterotypic tumor/immune cell-in-cell structures in tumor tissue arrays. (A): By using tumor tissue arrays containing sections representing tumor and paired adjacent tissues, tumor/immune cell-in-cell structures *in situ*. were calculated including liver cancer, cervical carcinoma and breast cancer. The percentage of heterotypic cell-in-cell structures was evaluated among 100 cells in one field of microscope. The average percentage from 5 randomly selected fields was used to represent the levels of cell-in-cell structure formation. (B): Representative images of heterotypic cell-in-cell structures in cervical carcinoma tissue arrays. Left: adjacent tissue section; Right: tumor tissue section (C): Representative images of heterotypic cell-in-cell structures in liver carcinoma tissue arrays. Left: adjacent tissue section; Right: tumor tissue section (D): Representative images of heterotypic cell-in-cell structures in breast cancer tissue arrays. Left: adjacent tissue section; Right: tumor tissue section Black arrows: heterotypic cell-in-cell structures Scale bar: 100 µM.

To further understand the physiological significance of heterotypic tumor/immune cell-in-cell structures, we compared the formation of heterotypic cell-in-cell structures between tumor and adjacent normal specimen. By using tissue arrays containing sections representing tumor and paired adjacent tissues, we investigated tumor/immune cell-in-cell structures *in situ*. We have observed the existence of tumor/immune cell-in-cell structures in tumor regions under investigation, including liver cancer, cervical carcinoma and breast cancer. More importantly, when compared the frequency of heterotypic cell-in-cell structures between tumor and adjacent tissues, higher percentage of heterotypic cell-in-cell structure was present in all types of tumors tissues under investigation (Fig. 4A). For instance, 5.78±2.10/field of tumor cell population formed heterotypic cell-in-cell structures in tumor tissues while 2.62±1.09/field of cell population in adjacent tissue samples in cervical carcinoma (P = 0.0001) (Fig. 4B). Similar variation was observed in liver carcinoma (Tumor: 3.24±1.23/field; Adjacent: 2.36±1.20/field, P = 0.088, Fig. 4C) or breast cancer (Tumor: 7.42±2.45/field; Adjacent: 2.54±1.03/field, P = 0.0000, Fig. 4D). Immune cells infiltrating tumor regions were internalized into tumor cells in a higher frequency, suggesting that tumor cells might adapt heterotypic cell-in-cell structure formation to limit the function and survival of immune cells. This might be beneficial for the adjacent tumor cells to escape from the attack of immune cells with better survival or proliferation of tumor cells.

### There Exhibit Diverse Forms of Cell-in-cell Structures after Internalization of Immune Cells into Tumor Cells

**Figure 5:**
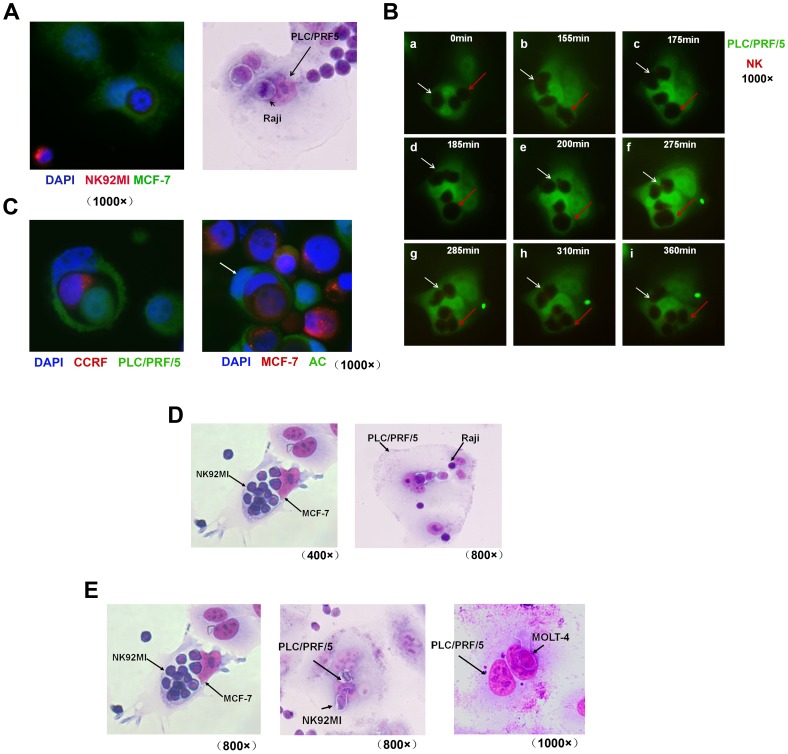
Diverse forms of internalized cells after the formation of heterotypic cell-in-cell structures. (A): Mitosis of NK92MI cells in MCF-7 (left) and Raji cells in PLC/PRF/5 (right). (B): Mitosis of NK92MI (labeled with CellTrack Red CMTPX) inside PLC/PRF/5 (labeled with CellTrack Green Bodipy) was tracked after heterotypic cell-in-cell structure formation by time-lapse microscope. Fluorescence and differential interference contrast (DIC) images were selected as the times indicated. NK92MI was indicated by arrows. (C): Left: PLC/PRF/5(green) was incubated with CCRF cells (red) for 4 hours followed by fixation. Tumor cell nucleus was labeled with DAPI. One PLC/PRF/5 (green) and one NK92MI cell (red) co-existed in PLC/PRF/5. Right: Heterotypic cell-in-cell structure by one MCF-7 and one AC cell were present inside another AC cell as white arrow indicated. (D): Multiple immune cells in one tumor cells. Left: NK92MI cells were incubated with MCF-7 for 4 hours. Right: Raji cells were incubated with PLC/PRF/5 for 4 hours. Cell mixture were fixed and stained with Giemsa dye. Dark staining indicated the nucleus of NK cells while tumor cell nucleus was slightly labeled. (E): Immune cells in the nucleus of tumor cells: MOLT-4 or N92MI were incubated with PLC/PRF/5 for 4 hours. Cell mixture was fixed and stained with Giemsa dye. Dark staining marked the nucleus of MOLT-4 or NK92MI while tumor cell nuclei were slightly labeled.

As we afore mentioned, heterotypic cell-in-cell structures were formed in a very common frequency. What is the fate of either host tumor cells or target immune cells? Our previous studies have described that when entering into tumor cells, most of NK92MI cells committed to apoptosis within it. But some of the internalized NK92MI cells underwent special biological behaviors inside tumor cell lines. For instance, internalized NK92MI cells or Raji cells underwent mitosis inside MCF-7 (Fig. 5A left) or PLC/PRF5 (Fig. 5A right), respectively. To track the mitosis of internalized cells, we have continuously observed the mitosis of NK92MI cells inside by using time-lapse microscope at the duration of 60 min (Movie S1). Looking closely at individual cells, three NK cells penetrated into one PLC/PRF/5 (Fig. 5B–a). One NK cell (white arrow) underwent mitosis first (Fig. 5B–b to e). Later on another NK (red arrow) underwent mitosis (Fig. 5B–f to h). This was quite unexpected. As immune cells and tumor cells are two rival parts for their own survival, the co-survival of these two cells is similar to the phenomenon of commensalism. The biological significance and mechanisms still need to be investigated.

Heterotypic cell-in-cell structures exhibited other forms. As shown in Fig. 5C, both immune cells (CCRF, red) and homotypic tumor cells (PLC/PRF/5, green) co-existed in tumor cells (left). Tumor cells, in some cases, internalized heterotypic cell-in-cell structure to form another type of heterotypic structure (right, indicated by white arrow). In addition, heterotypic cell-in-cell structure was formed by multiple immune cells inside one tumor cells. For instance, multiple NK92MI cells sojourned in one MCF-7 cells (Fig. 5D, left) while several Raji cells in one PLC/PRF5 cells (Fig. 5D, right). This might be occurred either through the mitosis of immune cells or the entrance of multiple immune cells. We have also observed that immune cells such as Molt-4 or NK92MI were internalized directly into the nuclei of PLC/PRF/5 host cells (Fig. 5E).

### Heterotypic Cell-in-cell Structures Lead to the Formation of Multinucleation with High Potency of Aneuploidy

**Figure 6:**
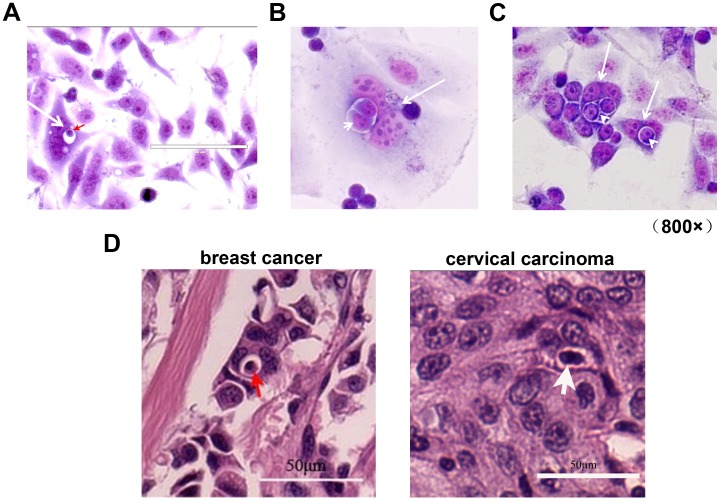
Multinucleation in heterotypic cell-in-cell structures. (A): Heterotypic cell-in-cell structure formation was performed through incubation of Chang liver and LAK cells for four hours. Chang liver cells (white arrow) were undergoing mitosis while one LAK cell (red arrow) penetrated into the nucleus of Chang liver cells. (B): MOLT-4 cells were incubated with PLC/PRF/5 cells for 4 hours following fixation and staining with Giemsa dye. NK92MI cells (arrowhead) underwent mitosis in PLC/PRF/5 (arrow). (C): MOLT-4 cells were incubated with Chang liver cells for 4 hours following fixation and staining with Giemsa dye. Several MOLT-4 cells (arrowhead) were penetrated into one Chang liver cell (arrow) as indicated. (D): Multinucleation in *in situ* tumorous regions of breast cancer and cervical cancer (arrow) as indicated. Scale bar: 50 µM.

Multinucleation is considered to be one of the mechanisms to form aneuploidy that is commonly observed in human tumors. It is demonstrated that the formation of homotypic cell-in-cell structure prevents the mitosis and induces the formation of aneuploid cells [6]. In our study, we have calculated the formation of multinucleation by using NK92MI cell lines as effector cells and tumor cells lines including A431, MCF-7 and PLC/PRF/5 as well as normal Chang liver cells as target cells. As listed in Table 3, we found that all four cell lines formed multinucleation when engulfing NK92MI cells with similar percentage. When observing precisely the morphology of multinuclear cell-in-cell structure, we found that the entrance of either one (Fig. 6A) or more (Fig. 6B and 6C) lymphocytes perturbed the formation of contractile ring during host cell division. This will probably lead to the caught of normal mitosis and the induction of numerical chromosomal aberrations. In the *in situ* tumorous region, we have also observed the multinucleation mediated by the infiltration of immune cells in tumors (Fig. 6D). Our results suggested that not only homotypic but also heterotypic cell-in-cell structure formation resulted in the abnormal mitosis and generation of potential aneuploid host cells.

**Table 3:** Percentage of polynuclear tumor cells in heterotypic cell-in-cell structures.

Target cell	Effector cell	Percentage of polynucleartumor cells in total	Percentage of polynucleartumor cells in cell-in-cellstructures
MCF-7	NK92MI	1.5%	21%
A431	NK92MI	1.5%	22.5%
PLC/PRF/5	NK92MI	6%	31.5%
Chang liver	NK92MI	1%	28%

## Discussion

In the present study, we have performed a large scale of survey for different types of cells to form heterotypic cell-in-cell structure with immune cells *in vitro* and *in vivo*. Our results revealed that unlike entosis occurring in the limited tumor cell lines, heterotypic cell-in-cell structure was formed *in vitro* by a large panel of cell lines such as tumor cell lines, normal tissues-derived cell lines as well as poorly differentiated stem cells with different efficacy. Several lineages of immune cells including T, B or NK cell lines were engaged in this active process. Moreover, heterotypic cell-in-cell structures were observed more distinguishably in tumor tissues compared to the adjacent regions, as well as in inflammatory regions. After internalization into host cells, the immune cells rested either in cytosol or nucleus. Some of them underwent mitosis inside. We have also observed the presence of multinucleation of host cells with the entrance of immune cells. Taken our observations together, it is obvious that heterotypic cell-in-cell structures belong to a unique mode of cell-cell interaction. As a active biological process, this interaction between tissue and immune cells not only changes the fate of the invaded and host cells, but also might alter the microenvironment where it happens.

Whereas the exact mechanisms initiating heterotypic cell-in-cell structure formation remains to be explored, the data presented here provide the direct evidence to demonstrate the extensiveness of heterotypic cell-in-cell structure at cellular and tissue levels. But it is unexpected that in our study the types of cell line to undergo homotypic cell-in-cell structure formation is dramatically less than those to perform heterotypic tumor-immune cell-in-cell structure formation *in vitro*. Through our survey, we found that most of cell lines tested could not perform entosis (Table 1). But heterotypic cell-in-cell structure formation occurs more frequently. Among cell lines with no entosis, part of them such as SK-BR-3, MDA-MB-231, PC-3, HepG2 *etc*. still underwent tumor/immune cell-in-cell structure formation with different frequency. Among tumor cell lines undergoing homotypic and heterotypic cell-in-cell structure formation, the higher frequency the entosis occurs, the higher ratio of heterotypic cell-in-cell structures happens (Table 1). We also found that tumor cells reached the highest frequency of heterotypic cell-in-cell structures between 4–8 hrs, which was similar to tumor cell lines undergoing entosis (data not shown). This suggested that tumor cells might undergo heterotypic cell-in-cell structure formation in a priority way. Considering the infiltration of lymphocytes in tumor regions *in vivo*, the priority to heterotypic cell-in-cell structure formation we observed *in vitro* suggests that tumor cells undergo distinct mechanisms to modulate the microenvironment.

While homotypic cell internalization probably results in the proper orchestration of multicellular organisms, the formation of heterotypic tissue/immune cell-in-cell structures might represent another biological significance under different conditions. During T cell development, it is observed that cytoplasmic vacuoles of thymic epithelial cells expressing keratin contained many completely internalized thymic cells, ranging in number from approximately 7 to 50 cells. These thymic epithelial cells were named as thymic nurse cells (TNCs), representing the most typical case of living cells internalizing other living cells and internalized cells being released from the host cell’s cytoplasm later on [24]. Through a “face transplant surgery” driven by MHC inside the TNCs, these internalized immature T cells differentiate and develop into T cells with mature surface markers before escaping from TNCs [25]. Benseler et al. recently found that when adoptively transferred into host animals, autoreactive T cells were condensed in the liver and internalized into liver epithelial cells. They quickly died through a cell-in-cell death pathway where lysosome participated in the process. If these T cells were blocked from internalization, the cellularity significantly increased in the periphery and liver tissue, thereby causing autoimmune damage [26]. These results indicate the roles of heterotypic cell-in-cell structures on lymphocyte development, differentiation, regulation, and homeostasis. In some cases, the occurrence of heterotypic cell-in-cell structures has became a characteristic of diseases such as Rosai-Dorfman disease, chronic myeloproliferative diseases and some hematological diseases [27]–[28].

In our study, we have observed higher frequency of heterotypic cell-in-cell structures in inflammation and caner status. It is well-known that inflammation- related cancer is largely driven by the production of growth and angiogenic factors that promote cancer cell survival, implantation and growth [29]. During chronic inflammation, the long-term involvement of immune cells remodels the tissues through altering the local microenvironment, including polarization of immune cells or production of cytokines [30]. In our *in vitro* study, we found that the formation of heterotypic cell-in-cell structures induced polynuclear cell structures. As shown in Fig. 6C, one or more MOLT-4 cells (arrowhead) penetrated into one Chang liver cell (arrow) that disrupts the formation of the contractile ring during host cell division similar to what has been observed after homotypic cell internalization. This will lead to the failure of cytokinesis, inducing the generation of aneuploid cells. Our study thus suggests another non-genetic mechanism of cytokinesis defect through the heterotypic cell-in-cell structure formation. We will not exclude the possibility that the leakage of chromosomes from internalized cells into host cells after penetrating directly into host cell’s nucleus or fusion with host cells might contribute to polyploidy as well as chromosomal instability of host cells. With higher frequency of invasion of immune cells into host tissue cells, this provides more probability for malignancy of cells. Considering extremely strong association between tumor and inflammation [31], formation of heterotypic cell-in-cell structures in inflammatory regions might be another potential mechanism for tumorigenesis. More importantly, multinucleation after heterotypic cell-in-cell structure formation during chronic inflammation might become the “transformation fast-track” of normal cells, which will substantially promote etiology and progression of tumor.

It is observed that higher tendency of internalization of immune cells by host tissue cells occurs compared to homotypic cell internalization. This might be due to the fact that these two processes adopt different mechanisms for internalization. During homotypic cell-in-cell structure formation E-cadherin and β-catenin are required for internalization coupling with Rho-Rock signaling activity [4]. On the other hand, ICAM-1, CD62L, LFA-2 or ezrin are demonstrated to be engaged in heterotypic cell-in-cell structure formation [10]–[11]. In the present study, with the co-culture of A431 and NK92MI, both A431/A431 homotypic and A431/NK92MI heterotypic cell-in-cell structure formation occur simultaneously. It needs to be further investigated whether two cellular processes depend on the properties of cell lines. This might also shed light on the mechanisms for the high tendency of heterotypic cell-in-cell structure in *in situ* tumor tissues or under inflammation.

### Conclusion

Taken together, we have reported the diversity and complexity of heterotyptic cell-in-cell interaction both *in vitro* and *in vivo*. More significantly, when considering the data from higher frequency of heterotypic cell-in-cell structures under inflammation and the subsequent multinucleation, we suppose that this unique cellular behavior might become another bona fide “highway” for the transformation of normal cells during chronic inflammation. To further decipher its cellular and molecular mechanisms will enrich our knowledge how heterotypic cell-in-cell structures influence the tumorigensis as well as progression and prognosis of tumors.

## Supporting Information

Movie S1 **NK92MI cells undergo mitosis in one PLC/PRF/5 cell.**
(WMV)
